# Spatial Multiomics Reveal Insights Into ADC Efficacy

**DOI:** 10.1002/eji.70190

**Published:** 2026-05-04

**Authors:** Osman Goni, Niklas Klümper, Maria del Mar Muñiz Moreno, Markus Eckstein, Christoph Kuppe

**Affiliations:** ^1^ Division of Nephrology and Clinical Immunology Medical Faculty RWTH Aachen University Aachen Germany; ^2^ Department of Urology University Hospital Bonn Bonn Germany; ^3^ Institute of Pathology Universitätsklinikum Erlangen, Friedrich‐Alexander‐Universität Erlangen‐Nürnberg Erlangen Germany

**Keywords:** antibody–drug conjugates (ADCs), metastatic urothelial cancer, spatial‐multiomics, therapy resistance, tumor microenvironment

## Abstract

Antibody–drug conjugates (ADCs) have transformed the therapeutic landscape of solid tumors; however, responses remain heterogeneous and complex to predict. In addition, a growing number of multiple ADC targets are either approved or in late‐stage clinical development, such as NECTIN‐4, HER2, or TROP2 for metastatic urothelial cancer. Spatial multiomics—representing next‐generation methods that couple high‐plex RNA sequencing and multiplex protein imaging with precise *x*‐*y*‐*z* coordinates within tissues—offer a direct way to correlate (ADC) antigen expression, cell state information, and micro‐anatomical context with patient treatment outcomes. In this review, we highlight suitability and technological advancements in current spatial transcriptomics and proteomics approaches to decode modes of action and resistance to ADCs and extract biological insights, particularly in metastatic urothelial cancer—and propose an integrative framework that combines spatial readouts with machine and/or deep learning‐driven analytics to stratify patients, forecast on‐ and off‐target toxicities, and guide next‐generation linker–payload designs or combination therapies.

## Graphical abstract

Spatial multiomics profiling can reveal how tumor heterogeneity and microenvironment influence ADC efficacy. Combining spatial transcriptomics, proteomics, and AI‐based analysis improves the identification of tissue factors influencing therapy response. This integrative approach potentially enables prediction of response, resistance, and optimized patient selection, which we review here.

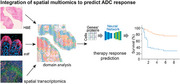

## 1 Introduction

Antibody–drug conjugates (ADCs) combine the target specificity of monoclonal antibodies with the cytotoxic potency of chemotherapeutic agents, offering a “guided missile” approach to treating tumors that express distinct surface antigens. As the first approvals in hematological malignancies—including, for example, Brentuximab Vedotin targeting CD30 in Hodgkin lymphoma [1]—ADCs have become established therapies in oncology. FDA approval of ADCs targeting NECTIN4 in metastatic urothelial cancer has propelled. In the Phase III EV‐302 trial, enfortumab vedotin plus pembrolizumab demonstrated a significant overall survival benefit compared to chemotherapy for patients with metastatic urothelial cancer [2].

However, the prediction of durable responses and resistance remains an open challenge. It is now well established across tumor types that ADC efficacy is closely linked to the expression level of the corresponding target antigen [3]. For enfortumab vedotin specifically, a correlation has been described between NECTIN4 expression or gene amplification and clinical response [4, 5]. Yet, even with a clear association between antigen expression and response, clinical outcomes remain variable, suggesting that additional spatial factors within the tumor microenvironment remain and critically influence the ADC therapeutic outcome. In this review, we discuss how spatial multiomics can be employed to reveal insights into ADC efficacy, determine the spatial distribution of ADC targets, and predict or monitor ADC responses with different tumor entities, including urothelial carcinoma, as a case study.

### 1.1 The Spatial Architecture of the Tumor Microenvironment

Tumors are composed of a mosaic of malignant cells, cancer‐associated fibroblasts, endothelial cells, myeloid populations, and diverse T‐ and B‐cell subsets [6]. Each compartment establishes metabolic and immunological niches that can potentiate or reduce ADC activity. A study revealed that even within a single biopsy core, NECTIN4 or HER2 expression can change from supra‐physiological to undetectable levels over a distance of <300 µm, often tracking histological substructures such as invasive fronts or hypoxic areas [7]. Cancer‐associated fibroblast‐rich areas remodel the extracellular matrix and increase lymphovascular invasion in bladder cancer [8]; such dense stroma may physically impede ADC diffusion. In this context, it is particularly striking that trastuzumab deruxtecan, a highly effective anti‐HER2 ADC that recently gained first tumor‐agnostic approval for metastatic solid tumors [3], demonstrates markedly limited efficacy in pancreatic ductal adenocarcinoma—a malignancy notoriously characterized by dense stromal architecture and elevated interstitial pressure that severely hinders drug penetrance [9]. Similarly, in non‐small cell lung cancer, immune‐rich regions with expression of PD‐L1 and IDO1 are separated from the surrounding stroma, creating spatial barriers that may reduce ADC efficacy [10]. Conversely, immune “hot islands” enriched for B cells and stem‐like PD‐1+ CD8 T cells have been linked to improved checkpoint blockade efficacy in urogenital tumors, raising the possibility that combined ADCs with immune checkpoint inhibitor regimens could synergize if spatial proximity is maximized and targetable in the tumor [11]. These spatial patterns raise a critical question: How can we decode and overcome these resistance mechanisms that limit ADC efficacy? Spatial transcriptomics, multiplexed imaging, and in situ proteomics enable high‐resolution mapping of ADC target expression, immune cell infiltration, extracellular matrix remodeling signatures, and stromal architecture within the tumor microenvironment. Ultimately, these insights may enable the conversion of resistance mechanisms into an opportunity for optimized and targeted ADC development.

### 1.2 Spatial Technologies to Decode the TME

Spatial multiomics profiling is currently more widely used to decode the tumor microenvironment [12]. Importantly, one needs to acknowledge and map each technology to the analytical blind spots it solves—from antigen patchiness to stromal barriers and bystander payload diffusion—which are precisely the variables that determine whether an ADC binds, internalizes, and kills the target cell [12].

**FIGURE 1:**
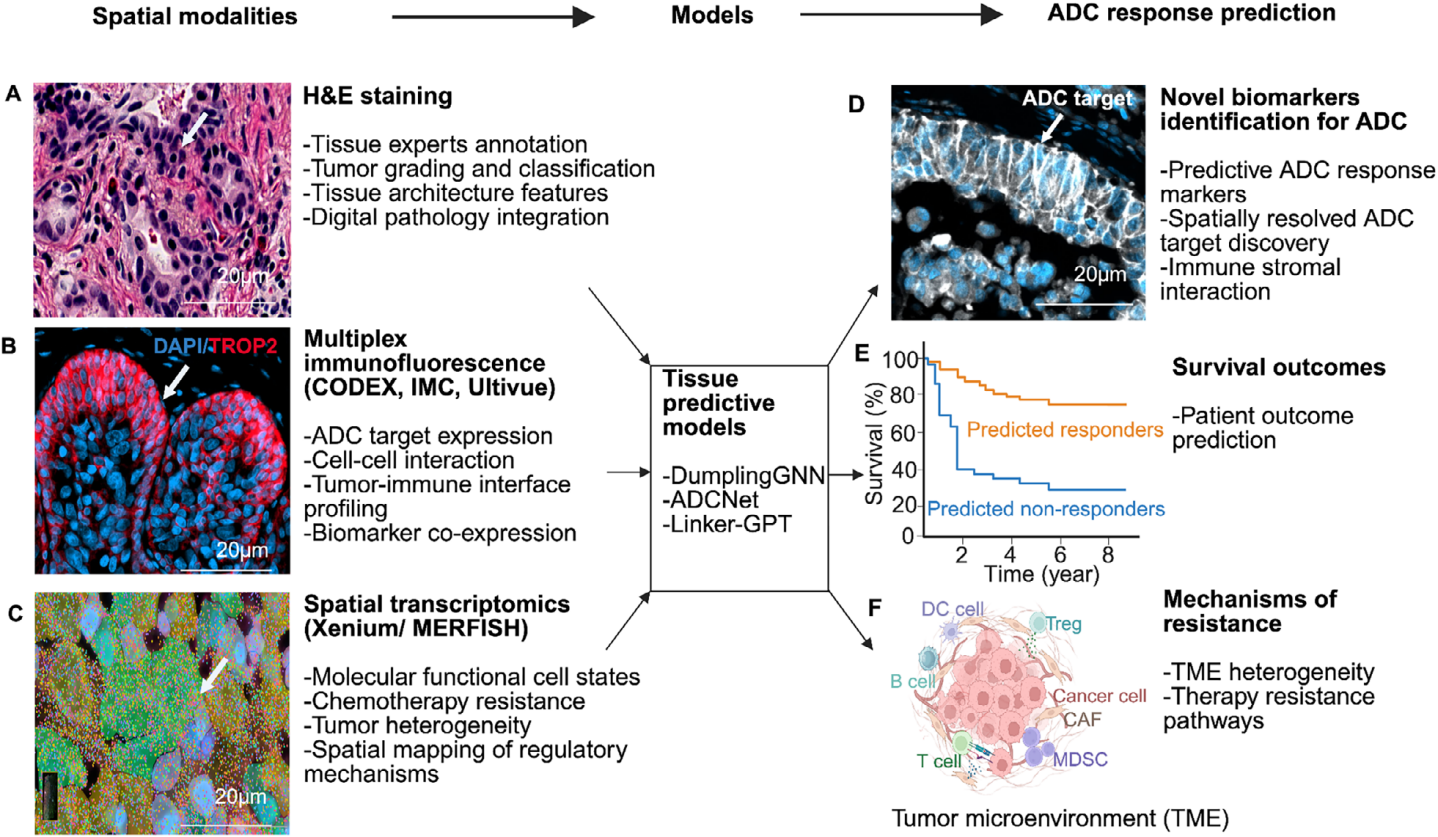
Integration of spatial multiomics using predictive modeling for antibody–drug conjugate (ADC) response prediction. (A) Hematoxylin and eosin (H&E) staining provides a histological context for tumor classification and guides digital pathology integration. (B) Multiplex immunofluorescence, including CODEX, imaging mass cytometry (IMC), Ultivue, enables ADC target expression analysis on a subcellular level (membranous vs. intracellular). (C) Spatial transcriptomics analysis captures gene expression heterogeneity, revealing resistant niches and regulatory pathways. (D) The tissue predictive model spatial features to identify novel ADC biomarkers based on the local expression patterns. (E) Spatially integrated models enable the prediction of patient responses. (F) Spatial models can further decode novel resistance mechanisms within the tumor microenvironment. Created in BioRender. https://BioRender.com/rq1m100. CODEX, co‐detection by indexing; MERFISH, multiplexed error‐robust fluorescence in situ hybridization.

CODEX (co‐detection by indexing) [13, 14] represents one of the high‐plex, low‐background multiplex imaging technologies of fluorescence multiplex imaging. A 56‐marker panel applied to cutaneous T‐cell lymphoma tissue illustrates the power of this approach, capturing structural, immune, and tumor markers without appreciable autofluorescence or spectral bleed‐through [15]. CODEX commonly utilizes heat‐induced epitope retrieval to unmask antigens, with variations in heating duration and temperature contributing to differences in staining outcomes (Table 1). Additionally, staining can be affected by batch effects resulting from variations in tissue fixation. Because CODEX resolves single‐cell contacts, it can also reveal whether an antigen‐bright niche is physically reachable by cytotoxic T cells that might synergize with the payload (Figure 1B).

**TABLE 1:** Overview of the multiplex platforms, including spatial resolution, modalities, advantages, disadvantages, and antibody–drug conjugate (ADC) studies.

Platforms	Modality	Spatial resolution	Advantages	Disadvantages	ADC studies
**CODEX**	Protein (metal‐tag antibodies)	∼1 µm, single‐cell	High multiplexing; quantitative, minimal spectral overlap	ROI‐based, relatively slow acquisition	Mapping ADC targets (e.g., NECTIN4, HER2 [14]
**Imaging mass cytometry (IMC)**	Protein (cyclic immunofluorescence)	Single‐cell	High‐plex workflow with preserved morphology	Long cycle times; antibody validation required	Spatial co‐expression of multiple ADC targets [30]
**Ultivue**	Protein (DNA‐barcoded antibodies)	Single‐cell	Simple workflow; reduced autofluorescence	Lower plex compared to CODEX/IMC	Rapid target and immune profiling for ADC stratification [21]
**Xenium**	RNA (in situ transcriptomics)	Subcellular	High sensitivity; subcellular resolution	Costly; limited protein readout	Mapping transcript‐level heterogeneity of ADC targets [25]
**MERFISH**	RNA (in situ transcriptomics)	subcellular	Ultra‐high plex, subcellular localization of transcripts	Limited throughput for large tissue sections	Identifying pathway‐level factors of ADC response [29]

Abbreviations: CODEX, co‐detection by indexing; MERFISH, multiplexed error‐robust fluorescence in situ hybridization.

Imaging mass cytometry (IMC) [16], which has recently been performed in 3D using cancer tissues [17], eliminates optical background entirely by replacing fluorophores with metal isotopes, which are quantified by time‐of‐flight mass spectrometry (Table 1). In lung adenocarcinoma, a 35‐plex IMC panel mapped more than 1.6 million cells and linked neighborhood signatures to clinical outcome [18, 19]. For ADCs, the same principle extends beyond phenotyping: Metal‐tagged antibodies against γH2AX or cleaved PARP can be multiplexed with the target antigen to show exactly where the linker has released its payload and how far the DNA‐damage niche spreads (Figure 1B). This makes IMC the experiment of choice for measuring bystander readouts or early resistance events such as up‐regulated DNA repair signatures.

Other multiplex technologies like InSituPlex from Ultivue close the loop between discovery and pathology workflows. Here, cocktails of four to 12 antibodies, each conjugated to a unique DNA tag, are revealed by complementary barcodes in sequential, non‐stripping cycles that fit on standard autostainers and slide scanners (Table 1). Validation across human and mouse tumors shows <20% deviation from single‐plex immunohistochemistry for each marker, and automated gating keeps inter‐run CVs below 25% [20, 21]. When combined with OmniVUE, the same workflow can escalate from four‐plex diagnostic panels to 12‐plex exploratory sets without changing hardware. Ultivue enables high‐plex detection of ADC targets and provides insights into antigen co‐localization, target accessibility, and spatial immune suppression that influence ADC efficacy (Figure 1B). This platform has been applied in the context of HER2 and TROP2‐directed ADCs to map target distribution and immune contexture in situ [22].

Xenium in situ transcriptomics provides subcellular RNA resolution for formalin‐fixed tissue [23]. Padlock probes hybridize to 300–5000 pre‐selected genes, undergo rolling‐circle amplification, and are read by 15 rapid imaging cycles, anchoring each transcript within ∼200 nm of its true location (Table 1). Furthermore, Xenium can be readily combined with hematoxylin and eosin staining (H&E staining), enabling the application of foundation models generated from H&E stainings, like the UNI model, to extract features not captured on the RNA level [24, 25]. Thus, Xenium enables the spatial measurement of ADC targets such as NECTIN4 and HER2, but also profiling the immune and stromal compartments on the protein level using the post‐Xenium section, which is amenable to further multiplex immunofluorescence stainings (Figure 1A). Furthermore, it can be combined with single‐nuclei RNA measurements from the same tissue block, which allows for higher resolution cell‐type annotation of the spatial data (Figure 1C) [26].

MERFISH [27]—multiplexed error‐robust fluorescence in situ hybridization—pushes transcript throughput an order of magnitude higher by assigning each RNA a binary barcode decoded over dozens of hybridization rounds (Table 1). Particularly interesting for ADC studies are the multiomics capabilities of combining RNA, protein, and even DNA readouts in one measurement at high resolution (Figure 1C) [28, 29]. Furthermore, gene panels can be fully customized between 140 and 1000 target transcripts.

In summary, these five platforms form a complementary toolbox. Multiplex protein assays like CODEX and IMC combine protein phenotyping with minimal optical noise; Xenium and MERFISH dissect transcriptional programs and emergent resistance with single‐molecule precision; Ultivue translates discoveries into pathology‐grade assays for reduced costs and higher throughput. In the future, merging such data streams with graph‐based AI to generate spatial response models and scores holds the promise to optimize patient therapy.

### 1.3 ADC Case Studies in Urothelial Cancer

In a previous study, we focused on the pattern of Nectin‐4 expression and linked the membranous expression of primary and patient‐matched distant metastases to clinical outcome (Table 2) [5]. Membranous Nectin‐4 expression was frequently decreased or absent in metastatic urothelial tissue sites, whereas the clinical benefit of enfortumab vedotin strongly depends on this membranous Nectin‐4 expression (Table 2). This underscores the importance of multiplex protein staining and their ability to measure subcellular protein distribution, as they likely define patient therapy responses. The amplification of Nectin‐4 represents another level of therapeutic response regulation due to copy number variation—a genomic alteration involving amplification or deletion of DNA, which we assessed on the mRNA level using FISH [4]. We demonstrated that Nectin‐4 amplifications are genomic predictors of enfortumab vedotin responses and long‐term survival in patients with mUC (Table 2). NECTIN4 itself is lowly expressed on mRNA level; thus, to quantify the expression level, a sensitive and specific approach is needed. Several factors have to be evaluated in this context, as recently also investigated here [35]. In retrospective analysis of UNITE, a multi‐institutional study, clinical outcomes of enfortumab vedotin in patients with urothelial carcinoma and various histologic subtypes were evaluated. Enfortumab vedotin showed meaningful responses in multiple variant histologies—including sarcomatoid and plasmatoid tumors, but its efficacy declined with increasing percentage of histologic subtypes. This finding suggests potential resistance in the certain subgroups and the need for spatial profiling to understand how histologic composition influences ADC efficacy [36].

**TABLE 2:** Clinical studies associated with the patient outcomes targeting biomarkers and target assessment.

Study	Targeted biomarker	Findings	Patient outcome	Target assessment	Clinical cohort
Klümper et al. [4]	NECTIN‐4	NECTIN‐4 amplifications are predictors of enfortumab vedotin response	96% of patients with NECTIN‐4 amplifications demonstrated an objective response rate to enfortumab vedotin compared with 32% in the non‐amplified subgroup	DNA: Genomic copy number analysis defining amplification	Metastatic urothelial cancer patients (*n* = 108)
Klümper et al. [5]	NECTIN‐4	NECTIN‐4 expression decreased in metastatic tumors relative to primary tumors (39.4% reduction in *H*‐score)	Reduced NECTIN‐4 expression was associated with significantly shorter progression‐free response on enfortumab vedotin; low expression predicts resistance	IHC H‐score comparison in primary and metastatic tumors	Primary metastatic paired urothelial cancer patients
Meric‐Bernstam et al. [3]	HER2	An increased survival benefit was observed for the high HER2 expression (IHC 3+) patients	Objective response rate and duration of response were 61.3% and 22.1 months in IHC 3+ compared with 37.1% and 11.3 months in all patients	Protein: HER2 expression assessed by IHC	Solid tumors, including breast and gastric cancer (*n* = 267)
Mosele et al. [31]	HER2	Trastuzumab deruxtecan provided clinical benefits with HER2‐overexpressing (IHC 3+), low (IHC 2+), over non‐expressing (IHC 0) patients	Objective response rate was observed 70.6% in HER2‐overexpressing patients, 37.5% in HER2‐low, and 29.7% in HER2‐non‐expressing patients	Protein: Objective response correlated with expression intensity	Phase 2 DAISY trial, metastatic breast cancer (*n* = 177)
Modi et al. [32]	HER2	Transtuzumab deruxtecan significantly improved compared with chemotherapy in patients with low expression of HER2 (IHC 1+)	Progression‐free survival and overall survival were 9.9 and 23.4 months, respectively, compared with 5.1 and 16.8 months in patients receiving chemotherapy	Protein: HER2‐low expression defined by IHC: randomized comparison with chemotherapy	Phase 3 DESTINY trial, previously treated low‐advanced breast cancer patients (*n* = 557)
Camidge et al. [33]	c‐Met	Durable response was associated with c‐Met protein overexpression (IHC 3+)	Objective response rate and duration of response were 28.6% and 8.3% in c‐Met high compared to 22.9% and 7.2 months in c‐Met intermediate expression	Protein: c‐Met quantified by IHC; efficacy stratified by expression	Phase II LUMINOSITY trial, non‐small cell lung cancer (*n* = 172)
Moore et al. [34]	FOLR1	Mirvetuximab soravtansine showed a significant benefit over chemotherapy	Progression‐free survival and overall survival were 5.62 and 16.46 months, respectively, compared with 4.34 and 12.75 months in patients receiving chemotherapy	Protein: FOLR1 expression quantified by IHC; high‐expression population compared with chemotherapy	Phase 3 MIRASOL trial, platinum‐resistant ovarian cancer (*n* = 453)

*Note: H*‐score, staining intensity score [1, 2, 3].

Abbreviation: IHC, immunohistochemistry.

### 1.4 Predictive Modeling and Computational Integration

Spatial‐omics generates terabytes of multiplex images and barcoded reads per patient. Machine learning methods—graph neural networks, spatially aware factor analysis (e.g., GLUE, MOFA+), and multimodal transformers—now label cell types, quantify neighborhood interactions, and output composite “spatial scores” [37]. Multi‐layer architecture that links raw images to clinical endpoints via self‐supervised embeddings has been recently proposed [12]. Computational frameworks such as ADCNet use a generalized deep neural network model for ADC efficacy prediction (Figure 1). The model integrates protein features from both antigens and antibodies, along with the information about linker, payload, and drug antibody ratio values that are highly correlated with the ADC activities [38]. Dumpling GNN, a hybrid Graph Neural Network model, captures multi‐scale molecular features using both 2D and 3D structural information and provides information into structure–activity relationships to predict ADC payload toxicity and activity (Figure 1) [39]. Linker‐GPT is an AI generative model to accelerate ADC linker development (Figure 1). The model integrates transfer learning from large‐scale molecular datasets and reinforcement learning to accelerate the discovery and optimization of ADC linkers (Figure 1) [40]. Recent spatial profiling of ADC monotherapies, showing intratumoral target heterogeneity, immune cell positioning, and local pharmacokinetic–pharmacodynamic features, influences therapeutic response. Combined scores of ADC expression density on RNA or protein level, fibroblasts, and T‐cell proximity, combined with other factors, might help to establish a predictive “spatial‐enfortumab vedotin‐scores,” which could help identify patients amenable to combination therapies.

### 1.5 Challenges

Current slide‐based spatial transcriptomic methods are limited to 5–10 µm sections, which risks 3D signal noise in tightly packed tissues and underrepresenting, for example, micro‐metastases [41]. Batch effects across platforms, sparse count matrices, and variable cell densities and pixel intensities complicate cross‐study harmonization. Community benchmarks [42] and federated learning are needed to calibrate spatial scores. Automation (e.g., Xenium autostainers) and region‐of‐interest pre‐selection can significantly reduce costs yet are not robustly established.

## 2 Conclusion

Spatial multiomics has moved from descriptive atlas building to generating actionable data for optimizing patient therapies. By analyzing where ADC antigens, immune effectors, and payload effects converge in the tissue—or fail to—and characterizing the TME at the cellular and subcellular levels, these technologies are set to improve patient care. The next step to achieve this required studies that embed spatial readouts into novel biomarker discovery and employ AI models that convert spatial data into clinically actionable insights, for example, predicted ADC therapy response.

## Author Contributions

Christoph Kuppe conceptualized the manuscript. Osman Goni and Christoph Kuppe wrote the original draft. Niklas Klümper, Maria del Mar Muñiz Moreno, and Markus Eckstein provided critical comments and supported with manuscript editing. Osman Goni and Christoph Kuppe prepared the figures and table of contents and finalized the manuscript. All authors read and approved the final manuscript.

## Funding

C.K. and M.E. received funding from the Else Kröner‐Fresenius‐Foundation (EKFS) for a clinician scientist professorship. N.K., M.E., and C.K. received funding from the Wilhelm Sander Foundation (Nr. 2024.022.1, DECODE‐ADC).

## Conflicts of Interest

The authors declare no conflicts of interest.

## Data Availability

No new data were generated in this review article.
